# Shared decision-making performance of general practice residents: an observational study combining observer, resident, and patient perspectives

**DOI:** 10.1093/fampra/cmad125

**Published:** 2024-01-11

**Authors:** Anouk Baghus, Esther Giroldi, Jasper van Geel, Arthur Leferink, Marjolein H J van de Pol, Ariëtte Sanders, Patrick W Dielissen, Isabella Bisschop, Arwen H Pieterse, Jean W M Muris, Angelique A Timmerman, Trudy van der Weijden

**Affiliations:** Department of Family Medicine, Care and Public Health Research Institute, Maastricht University, Maastricht, The Netherlands; Department of Family Medicine, Care and Public Health Research Institute, Maastricht University, Maastricht, The Netherlands; Department of Educational Development and Research, School of Professional Education, Maastricht University, Maastricht, The Netherlands; Department of Family Medicine, Care and Public Health Research Institute, Maastricht University, Maastricht, The Netherlands; Department of Family Medicine, Care and Public Health Research Institute, Maastricht University, Maastricht, The Netherlands; Department of Primary and Community Care, Radboud University Medical Center, Nijmegen, The Netherlands; Julius Centre for Health Sciences and Primary Care, University Medical Centre Utrecht, Utrecht, The Netherlands; Department of Primary and Community Care, Radboud University Medical Center, Nijmegen, The Netherlands; Department of Public Health and Primary Care, Leiden University Medical Center, Leiden, The Netherlands; Medical Decision Making, Department of Biomedical Data Sciences, Leiden University Medical Center, Leiden, The Netherlands; Department of Family Medicine, Care and Public Health Research Institute, Maastricht University, Maastricht, The Netherlands; Department of Family Medicine, Care and Public Health Research Institute, Maastricht University, Maastricht, The Netherlands; Department of Family Medicine, Care and Public Health Research Institute, Maastricht University, Maastricht, The Netherlands

**Keywords:** decision making, education, general practice, graduate, medical, observational study, patient involvement, patient-centred care, shared

## Abstract

**Background:**

Shared decision making (SDM) is considered fundamental to person-centred care. However, applying SDM may be a challenge for residents in general practice, since it is a complex competence that requires the integration of knowledge and skills from several competency domains.

**Objective:**

To support learning of SDM during medical residency, we aimed to gain insight in Dutch residents’ observed and perceived SDM performance in general practice.

**Methods:**

We evaluated residents’ SDM performance from an observer, resident, and patient perspective. Consultations of first- and third-year residents were recorded. Trained observers used the validated Observing Patient Involvement (OPTION^5^) scale to assess observed SDM performance of residents in 98 actual recorded consultations. Perceived SDM performance was evaluated by residents and patients completing validated SDM questionnaires, supplemented with questions about (the context of) the consultation and perceived relevance of SDM immediately after the consultation. The data were analysed using descriptive statistics (mean, SD, minimums, and maximums) and explorative bivariate analyses.

**Results:**

The residents’ observed mean SDM performance was 19.1 (range, 0–100, SD = 10.9), mean resident self-reported SDM performance was 56.9 (range, 0–100, SD = 18.5), and mean patient-reported SDM performance was 73.3 (range, 0–100, SD = 26.8). We found a significant and positive correlation between observed SDM performance and residents’ perceived relevance of SDM for the consultation (*t* = 4.571, *P* ≤ 0.001) and the duration of the consultation (*r* = 0.390, *P* ≤ 0.001).

**Conclusions:**

This study showed that there is room for increasing awareness of the potential incongruence between observed and perceived SDM performance during medical residency, in order to facilitate the implementation of SDM in clinical practice.

**Key messages**

- There is incongruence between residents’ perceived and observed SDM performance.
- More SDM is performed when residents consider consultations relevant for SDM.
- Focus on SDM during medical residency is needed for application in clinical practice.

## Background

Person-centred care is an important core value of general practice,^1,2^ as the focus is on the patient as a person in their individual context, rather than solely on the disease.^3^ Shared decision making (SDM) is considered fundamental in person-centred care.^3,4^ In SDM, the clinician and patient work together to make a deliberate decision based on the clinician’s expertise and the patient’s values and preferences, combined with the available scientific evidence.^4,5^

SDM may be considered challenging, especially for young clinicians, as it requires an integration of several competency domains, such as communication, medical expertise, collaboration, and scholarship in the clinical encounter.^6–8^ Yet, experienced clinicians are often unaware of potential SDM incompetence, as in their perception, they already involve patients in decision making, although the occurrence of SDM during encounters is limited.^9–11^ This suggests that current SDM training is still insufficiently tailored to the needs of future clinicians. As the focus is on clinical workplace learning during medical residency, SDM competency is expected to be developed specifically in this period.^12–14^ However, multiple challenges are faced in learning during medical residency. These challenges include residents’ perceived limited medical knowledge of the options at stake, strong personal preferences in the decision being made, lack of good role models, and residents’ cognitions on patients’ ability and motivation to be involved in decision making.^6,15–17^

To support learning of SDM during medical residency, we need to get insight into the SDM performance of residents, as the current SDM performance of residents is yet unknown. To capture the complexity of the decision-making process, evaluation of observed performance and experiences during the clinical encounter are relevant.^18,19^ The research questions of this study were: (i) what is the observed SDM performance of residents?; (ii) how do residents themselves and their patients perceive the application of SDM?; and (iii) how does the observed residents’ performance relate to their self-perception of SDM application during the encounter?

## Methods

### Study design

We conducted a quantitative descriptive study using recorded consultations and questionnaires, which focused on observations and perceptions of the decision-making process during these consultations.

### Setting and participants

This study was conducted with residents from four General Practice (GP) training institutes in the Netherlands. During their first and third year of the 3-year GP specialty training, residents work in general practice. GP supervisors are available to provide direct supervision when indicated. A characteristic of specialty training is that residents mainly learn through workplace learning, where they gain increasing autonomy and responsibility for increasingly complicated medical problems.^20^ In the formal curriculum of Dutch GP specialty training, SDM is mainly integrated in communication training as a separate skill (Supplementary Appendix A). This education focuses on providing theory and on practicing skills during a limited number of scheduled communication training sessions throughout specialty training.

First- and third-year GP residents of the Dutch GP training institutes in Maastricht, Amsterdam, Nijmegen, and Leiden were informed about the study aims and procedure during a training day at the GP training institute. Residents who were interested in participating received an information letter. Residents were aware that we studied their SDM performance level, although we did not specify which indicators were assessed. Participating residents were compensated with a €25 gift card. Patients received verbal and written information about “a study on doctor-patient communication” from one of the researchers in the waiting room of the general practice. If the patient was under 18, also the accompanying parent(s) were informed. Patients received no financial compensation for their participation.

### Data collection

One of the researchers visited the residents in their general practices during a morning or afternoon clinic between August 2017 and December 2019. During this clinic, the consultations were video-recorded. Whenever this was not possible due to technical reasons or objections of the patient, the consultation was audio-recorded. Patients were eligible if they had a good enough command of the Dutch or English language. Patients were not included or excluded based on the diagnosis or decision at stake. We collected the participants’ demographic characteristics after the encounter. Consultations were included if a decision was made.

### SDM measures

#### Shared decision-making questionnaires.

 For the evaluation of perceived SDM performance, the researcher provided residents and patients with questionnaires to fill out directly after the consultation. We used the following SDM questionnaires validated in Dutch: the SDM-Q-Doc (residents) and the SDM-Q-9 (patients).^21–23^ Both scales consist of nine items, which are rated on a six-point Likert scale (0, “strongly disagree”—5, “strongly agree”). In residents, the questionnaire was supplemented with questions about the context of the consultation—(un)known patient, emergency consultation, initial, or follow-up consultation,—the reason for encounter, and the decision being made. To measure how relevant residents and patients considered SDM for their consultation, we added the following question to be rated on the same six-point Likert scale: “How relevant was SDM in this consultation” (residents); “For me it was important to be involved in the decision made in this consultation” (patients).

#### Observing Patient Involvement (OPTION) scale

. After finishing data collection in general practice, the validated Observer OPTION^5^ scale was used to assess SDM performance in the recorded consultations from an observer perspective.^24,25^ This scale consists of five items to assess SDM performance on a five-point Likert scale (0: no effort—4: exemplary effort). These items are based on the conceptual framework of SDM, as described by Elwyn et al.^26^ Two observers independently scored the recorded consultations. The sequence of the consultations was randomized by using stratified randomization to minimize observer bias. In case more than one medical decision was made during the consultation, the main decision was scored.

Before scoring the consultations, the observers (AB, JvG) were trained using the English OPTION^5^ manual and coding sheet. The training was performed as follows: first, the research team (AB, EG, JvG, JM, AT, TvdW) read the manual thoroughly and then they independently scored five video-recorded consultations, as did a researcher experienced in using the OPTION^5^. All met to discuss the manual, the measure score sheet and scores. After 1 week, this process was repeated and the same five consultations were independently scored again. For calibration purposes, the two observers independently scored another five consultations after which they met to discuss their results and assessed inter-rater reliability (IRR) using an intra-class correlation coefficient (ICC). This process was repeated until an acceptable ICC of >0.6 was established.^24^ The ICC on the total OPTION^5^ score was 0.86, including an ICC >0.6 on all individual OPTION^5^ items. All consultations used for training and calibration were initially collected for another study and are, therefore, not part of the sample for the current study. We received permission to use these recordings for this purpose.

### Data analysis

The two observers included only consultations in which a medical decision was made since we regard SDM as relevant in this situation. Disagreements were discussed within the research team. The scores of the OPTION^5^ and questionnaires were digitized in SPSS 25 for Windows (IBM Corp., Armonk, New York). To ensure the accuracy of the data, all digitized data were double-checked. We calculated means per OPTION^5^ item scores from both observers and rescaled the total scores of OPTION^5^ (range 0–20) to a 0–100 scale according to the manual.^24^ To rescale the total scores of SDM-Q-Doc and SDM-Q-9 (range 0–45) to a 0–100 scale, we used means of individual item scores to replace missing values for up to two random missing values per consultation.^21^ In case of more than two missing individual item scores, the total score was not calculated. We used descriptive statistics to determine means, SD, minimums, and maximums for individual items and total scores. The total OPTION^5^, SDM-Q-Doc, and SDM-Q-9 scores were normally distributed. Therefore, parametric tests were used to explore associations between potential clarifying variables regarding residents (age, gender, training year, training institution, clinical experience), patients (age, gender, educational level), and consultations (relevance of SDM, perception of SDM, duration of the consultation, (un)known patient, initial or follow-up consultation and emergency consultation) and total OPTION^5^ score. We considered total OPTION^5^, SDM-Q-Doc, and SDM-Q-9 scores as continuous variables. We used Pearson’s correlation to explore associations between the total OPTION^5^ score and the continuous variables. We used an independent sample *t*-test for discrete potential clarifying variables categorized in two groups and one-way ANOVA for discrete potential clarifying variables categorized in more than two groups (Supplementary Appendix B).

### Ethical approval and informed consent

The Ethics Committee of the Dutch Association for Medical Education (NVMO) approved this study (file no. 894). Participating residents and patients gave written informed consent; GP supervisors gave verbal permission to visit the practice.

## Results

### Participant characteristics

**Fig. 1.**
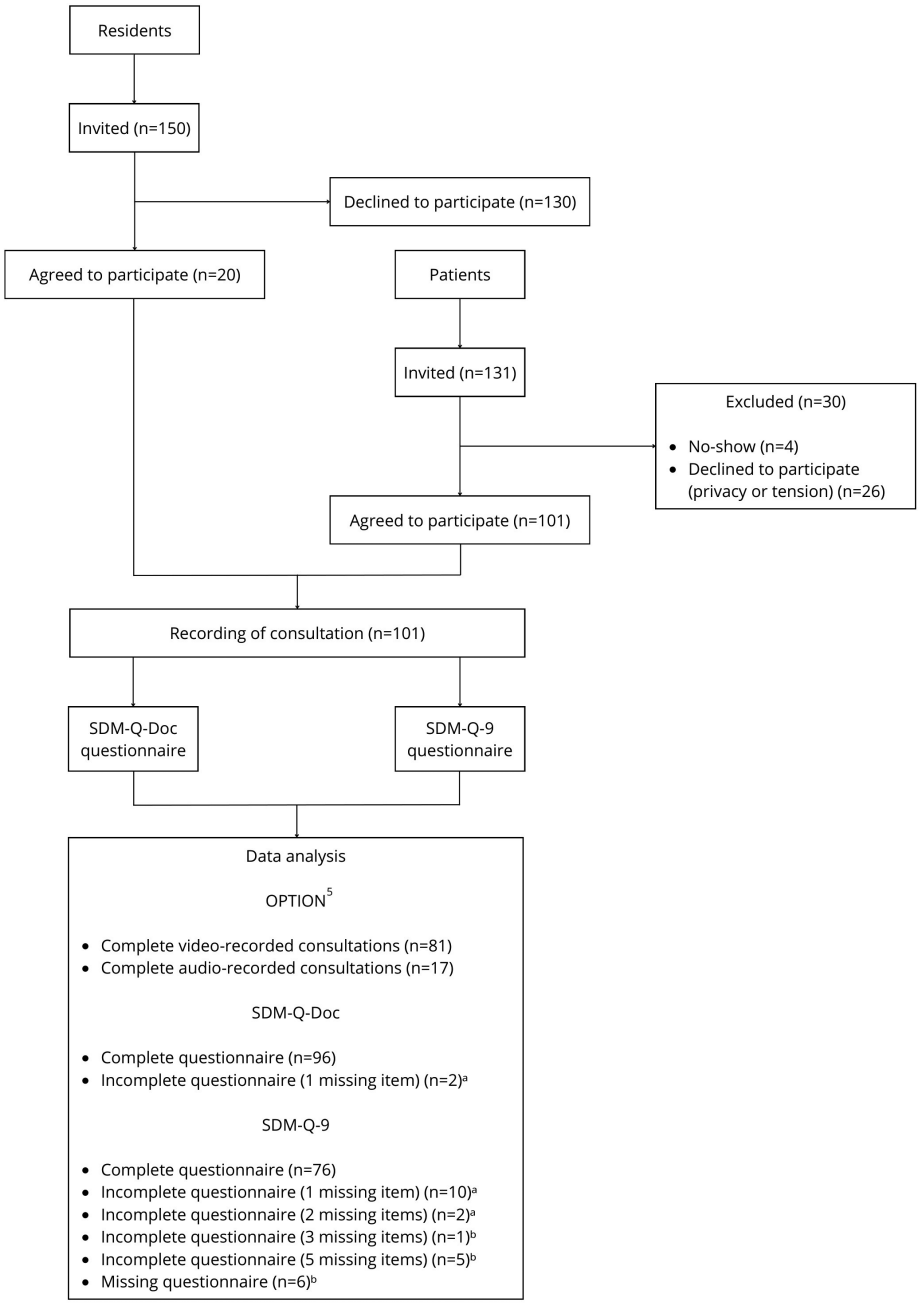
Flow-chart of the process of inclusion of participating general practice residents and patients, and the sources and number of data collected for a study of shared decision-making performance in medical residency, 2017–2019. Abbreviations: OPTION, observing patient involvement; SDM-Q-Doc, shared decision-making questionnaire filled in by residents; SDM-Q-9, shared decision-making questionnaire filled in by patients. ^a^These incomplete questionnaires are included in the total score of SDM-Q-Doc/SDM-Q-9. ^b^These incomplete questionnaires are not included in the total score of SDM-Q-9.

Of 150 GP residents invited, 20 agreed to participate in this study of which 11 were first-year residents and nine were third-year residents (see Table 1 and Supplementary Appendix C). Reasons for not participating included busy schedules and feeling uncomfortable with consultations being recorded and assessed. All residents had prior clinical experience before entering GP specialty training. From the 131 consultations scheduled at the clinics visited by the researchers, 101 patients agreed to participate (see Fig. 1). From the 101 consultations, 3 were not scored due to incomplete recordings. Of the remaining 98 recordings, 81 were video-, and 17 were audio recordings. All 98 consultations were included as there was a medical decision made in each consultation. Seventeen consultations concerned children visiting the resident accompanied by a parent. Most patients consulted because of musculoskeletal, respiratory, or skin complaints. The consultations had a mean duration of 13.8 min. The mean score of the perceived relevance of SDM for the consultations was 2.9 for residents and 4.3 for patients (range, 0–5).

**Table 1.** Characteristics of participating general practice residents (n = 20), patients (n = 98) and consultations (n = 98), for a study of shared decision-making performance in medical residency, 2017–2019.

Resident characteristic	*n* (%)^a^
**Age (mean in years [range])**	**30 [25–35]**
**Female**	**16 (80)**
**GP training year**	
First year	11 (55)
Third year	9 (45)
**Clinical experience before entering GP training**	
In hospital setting only	7 (35)
In non-hospital setting only	3 (15)
In both hospital and non-hospital setting	10 (50)
**Duration of clinical experience before entering GP training (mean in months [range])**	**31.7 [6–60]**
**Number of patient inclusions (mean [range])**	**4.9 [3–8]**
**General practice type**	
Urban	17 (85)
Rural	3 (15)
**Location of GP training institute**	
Maastricht	6 (30)
Amsterdam	5 (25)
Nijmegen	4 (20)
Leiden	5 (25)

Patient characteristic	*n* (%)^a^
**Age (mean in years [range])^b^^,^^c^**	**51 [18–85]**
**Adult patient**	**81 (83)**
**Female^b^**	**64 (65)**
**Educational level^b^^,^^c^^,^^d^**	
Low	26 (32)
Middle	27 (33)
High	22 (27)

Consultation characteristic	*n* (%)^a^
**Type of consultation**	
Unknown patient	57 (58)
Initial consultation	65 (66)
Emergency consultation	0 (0)
**Reason for encounter^e^**	
General and unspecified	3 (3)
Digestive	6 (6)
Eye	4 (4)
Ear	5 (5)
Cardiovascular	3 (3)
Musculoskeletal	16 (16)
Neurological	1 (1)
Psychological	5 (5)
Respiratory	23 (24)
Skin	24 (25)
Endocrine/metabolic and nutritional	4 (4)
Pregnancy, childbearing, family planning	3 (3)
Female genital	1 (1)
Male genital	1 (1)
**Nature of decision**	
Wait-and-see	20 (20)
Diagnostic test	20 (20)
Medication^f^	41 (42)
Referral^g^	12 (12)
Intervention^h^	5 (5)
**Relevance of SDM for the consultation**	
SDM relevance according to resident (mean (SD))	2.9 (1.3)
SDM relevance according to patient (mean (SD))^i^	4.3 (1.3)
**Duration of consultation (mean in minutes (SD))**	**13.8 (5.4)**

Abbreviations: GP = General Practice; SDM = shared decision making; SD = standard deviation.

^a^Data are n (%) unless otherwise indicated.

^b^Based on data of adult patients and accompanying parent of patient under 18.

^c^6 missing cases.

^d^Educational level of patients was grouped in low (no education, primary education, lower secondary education), middle (higher secondary education, technical/vocational further education) or high (bachelor, master).

^e^Classification of reason for encounter according to International Classification of Primary Care (ICPC).

^f^Decisions regarding starting, continuing or quitting medication, or adjustments to the doses.

^g^Decisions regarding referrals to primary care health care professions (e.g., physiotherapist) or hospital.

^h^Decisions regarding interventions in general practice (e.g., surgical, cryotherapy, bandages).

^i^10 missing cases.

### Level of SDM performance

#### Observed performance of SDM.

The inter-rater reliability on the total OPTION^5^ score was high (ICC, 0.87), including an ICC > 0.6 on all individual OPTION^5^ items. The mean total OPTION^5^ score for all 98 consultations was 19.1 (SD = 10.9). Table 2 shows the scores per OPTION^5^ item. Scores per OPTION^5^ item ranged from no effort to moderate or skilled effort. The lowest mean score was found for “supporting deliberation” (item 2), which means that on average residents put little effort into supporting patients in considering the decision options.

**Table 2.** Observed shared decision-making performance scores as assessed using OPTION^5^: individual item scores and total score for consultations, for a study of shared decision making-performance in medical residency, 2017–2019.

Observer OPTION^5^ per item, scale 0–4^a^	OPTION^5^ (*n* = 98)			
	Mean	SD	Minimum	Maximum
**Item 1.** For the health issue being discussed, the clinician draws attention to or confirms that alternate treatment or management options exist or that the need for a decision exists. If the patient rather than the clinician draws attention to the availability of options, the clinician responds by agreeing that the options need deliberation.	0.7	0.6	0	3
**Item 2.** The clinician reassures the patient or re-affirms that the clinician will support the patient to become informed or deliberate about the options. If the patient states that they have sought or obtained information prior to the encounter, the clinician supports such a deliberation process.	0.2	0.5	0	2
**Item 3.** The clinician gives information or checks understanding about the options that are considered reasonable (this can include taking no action), to support the patient in comparing alternatives. If the patient requests clarification, the clinician supports the process.	1.1	0.6	0	3
**Item 4.** The clinician makes an effort to elicit the patient’s preferences in response to the options that have been described. If the patient declares their preference(s), the clinician is supportive.	1.0	0.7	0	3
**Item 5.** The clinician makes an effort to integrate the patient’s elicited preferences as decisions are made. If the patient indicates how best to integrate their preferences as decisions are made, the clinician makes an effort to do so.	0.9	0.6	0	2.5

Observer OPTION^5^ total score, scale 0–100^b^	Mean	SD	Minimum	Maximum
Total score	19.1	10.9	2.5	55

Abbreviations: OPTION = Observing Patient Involvement; SD = standard deviation.

^a^Score on a scale of 0-4 (0 = no effort, 1 = minimal effort, 2 = moderate effort, 3 = skilled effort, 4 = exemplary effort).

^b^Score on a scale of 0-100, calculated as the sum of the five individual item mean scores and rescaled from 0-20 to 0-100.

#### Residents’ perceptions of SDM performance.

The mean total SDM-Q-Doc score was 56.9 (SD = 18.5), see Table 3. Item 4 (informing about the benefits and risks of the options) and item 7 (negotiation) were rated lowest by residents, while item 5 (investigation of patient’s understanding and expectations) and item 9 (arrangement of follow-up) received the highest scores.

**Table 3.** Residents’ and patients’ perceived shared decision-making performance scores as assessed using the SDM-Q-Doc and SDM-Q-9: item scores and total score for consultations, for a study of shared decision-making performance in medical residency, 2017–2019.

	SDM-Q-Doc (*n* = 98) Residents		SDM-Q-9 (*n* = 92) Patients	
SDM-Q-Doc / SDM-Q-9 per item, scale 0-5^a^	Mean (SD)	Missing (*n* (%))	Mean (SD)	Missing (*n* (%))
**Item 1.** I made clear to my patient that a decision needs to be made/My doctor made clear that a decision needs to be made	2.9 (1.2)	0 (0)	3.6 (1.7)	0 (0)
**Item 2.** I wanted to know exactly from my patient how he/she wants to be involved in making the decision/My doctor wanted to know exactly how I want to be involved in making the decision	2.6 (1.2)	0 (0)	3.5 (1.8)	0 (0)
**Item 3.** I told my patient that there are different options for treating his/her medical condition/My doctor told me that there are different options for treating my medical condition	2.7 (1.4)	1 (1.0)	3.6 (1.7)	2 (2.2)
**Item 4.** I precisely explained the advantages and disadvantages of the treatment options to my patient/My doctor precisely explained the advantages and disadvantages of the treatment options	2.3 (1.4)	0 (0)	3.3 (1.9)	6 (6.5)
**Item 5.** I helped my patient understand all the information/My doctor helped me understand all the information	3.3 (0.9)	0 (0)	4.4 (1.1)	9 (9.8)
**Item 6.** I asked my patient which treatment option he/she prefers/My doctor asked me which treatment option I prefer	2.9 (1.5)	0 (0)	3.6 (1.8)	8 (8.7)
**Item 7.** My patient and I thoroughly weighed the different treatment options/My doctor and I thoroughly weighed the different treatment options	2.3 (1.2)	1 (1.0)	3.3 (1.8)	10 (10.9)
**Item 8.** My patient and I selected a treatment option together/My doctor and I selected a treatment option together	3.0 (1.4)	0 (0)	3.8 (1.6)	7 (7.6)
**Item 9.** My patient and I reached an agreement on how to proceed/My doctor and I reached an agreement on how to proceed	3.7 (1.3)	0 (0)	4.0 (1.7)	6 (6.5)

SDM-Q-Doc/SDM-Q-9 total score, scale 0–100^b^	Mean (SD)	Missing (*n* (%))	Mean (SD)	Missing (*n* (%))
**Total score**	56.9 (18.5)	0 (0)	73.3 (26.8)	6 (6.5)^c^

Abbreviations: SDM-Q = shared decision-making questionnaire; SD = standard deviation.

^a^Score on a scale of 0-5 (0 = strongly disagree, 1 = disagree, 2 = more or less disagree, 3 = more or less agree, 4 = agree, 5 = strongly agree).

^b^Total score on a scale of 0-100, calculated as the sum of the nine individual item mean scores and rescaled from 0-45 to 0-100.

^c^In case of >2 missing individual item scores, the total score was not calculated.

#### Patients’ perceptions of SDM performance.

The mean total SDM-Q-9 score was 73.3 (SD = 26.8), see Table 3. Patients scored all items higher compared to the residents, yet their scores showed the same patterns. Patients also gave the lowest scores to items 4 and 7, and the highest scores to items 5 and 9.

#### Associations between observed and perceived SDM performance.

**Fig. 2.**
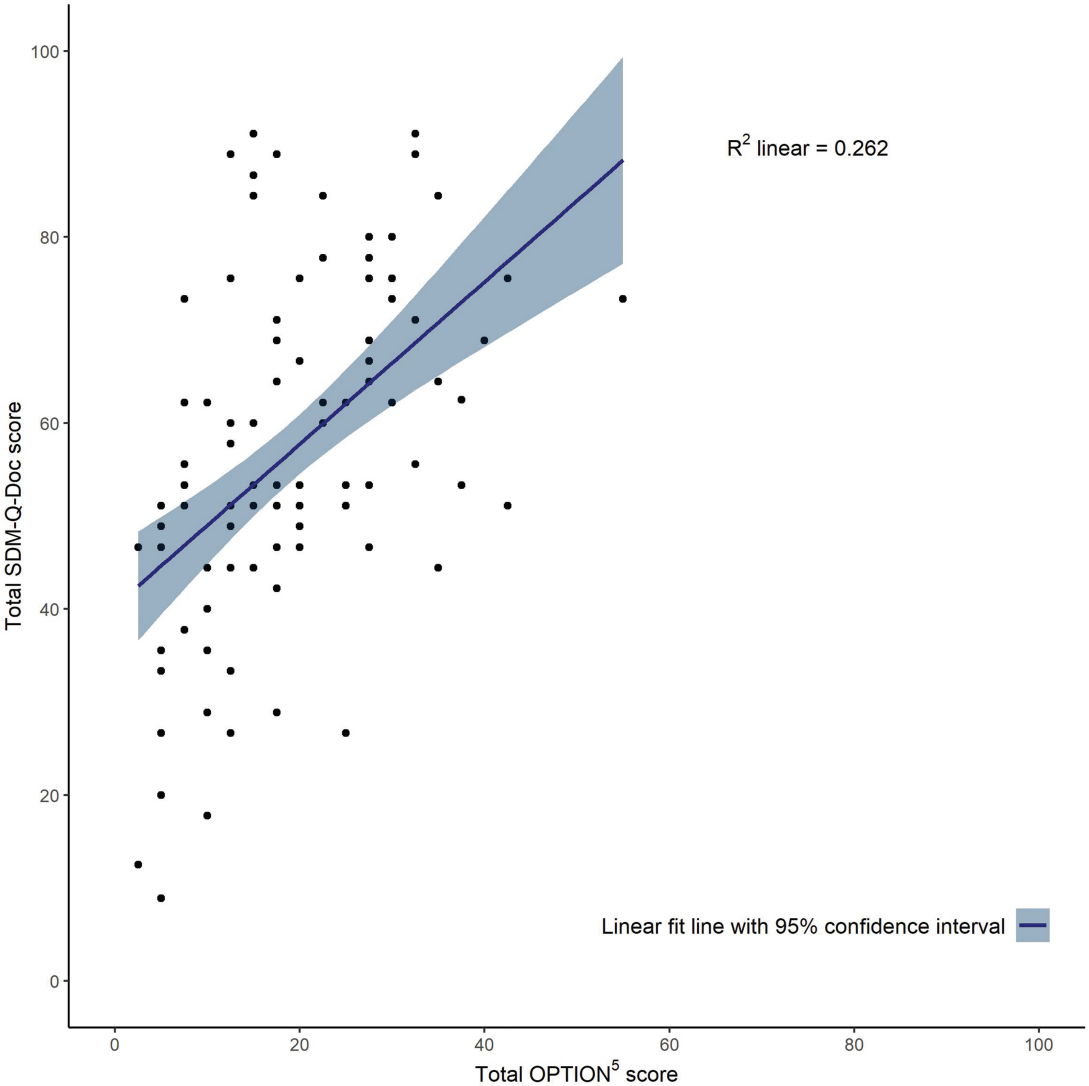
Scatter plot of observed shared decision-making performance (total OPTION^5^ scores) (*n* = 98) and residents’ perceptions of shared decision-making performance (total SDM-Q-Doc scores) (*n* = 98) for a study of shared decision-making performance in medical residency, 2017–2019. Abbreviations: OPTION = Observing Patient Involvement; SDM-Q-Doc = shared decision-making questionnaire filled in by residents

There was a significant positive correlation between the total OPTION^5^ score and residents’ perception (SDM-Q-Doc) (*r* = 0.512, *P* < 0.001), see Figure 2, and patients’ perception of SDM (SDM-Q-9) (*r* = 0.214, *P* = 0.048) (see Supplementary Appendix B).

#### Associations between observed SDM performance and resident, patient and consultation characteristics.

 OPTION^5^ scores of consultations in which residents found SDM relevant (score ≥3 on a six point Likert scale ranging from 0 to 5) were compared to scores of consultations in which residents found SDM not relevant (score <3). Observed performance was significantly higher (*P* ≤ 0.001) in consultations for which SDM was perceived to be more relevant (OPTION^5^ score 22.3) compared to consultations for which SDM was perceived to be less relevant (OPTION^5^ score 12.7). OPTION^5^ scores were also significantly and positively associated with the duration of the consultation (*r* = 0.390, *P* ≤ 0.001), which means that more SDM was observed in longer consultations.

## Discussion

This study aimed to describe both observed and perceived SDM performance of residents in GP specialty training. Observed SDM performance of residents was much lower compared to residents’ and patients’ perceptions of SDM performance. Observed SDM performance was somewhat higher if the resident felt SDM to be relevant in the consultation and if more time was spent during the consultation.

### Reflection on main findings

The overall OPTION^5^ score of 19.1 was lower than the benchmark of 25.^27,28^ Several explanations can be given for this finding. Residents may not be aware of what SDM actually entails and how to apply SDM in clinical practice. Besides, a high cognitive load is present in learning SDM as a complex skill that integrates medical knowledge and expertise, evidence based medicine and communication skills.^6,16^ Still, residents’ performance was slightly lower than SDM performance of experienced GPs in most studies.^27,29,30^ This may explain that experienced clinicians often perceive that they already involve patients in decision making, although the occurrence of SDM during encounters is limited.^9–11^ Therefore, clinical supervisors might not be able to be adequate role models and coaches in residents’ learning process of SDM at the workplace and to provide them with good examples of SDM in clinical encounters.

Since SDM requires context-specific application, it could be justifiable to perform less SDM in situations in which this is not relevant from a medical perspective. However, it is doubtful whether residents are able to recognize situations in which SDM is relevant. Residents’ beliefs in this respect are affected by multiple contextual factors (e.g. patient characteristics, clinical scenario and residents’ personal preferences regarding the options).^6,9,16^ They tend to consider SDM especially relevant for complex decisions with important consequences for patient and care (e.g. end-of-life, lifestyle, chronic disease), whereas for many decisions in general practice the complexity is relatively low (e.g. simple decisions on curative treatment for patients without co-morbidity or risk factors for complications).^16^ It is also notable that research showed that residents appear to prefer paternalistic decision making more often than experienced clinicians.^7^ Therefore, residents may very well underestimate the actual relevance of SDM, causing residents’ underperformance as observed in this study. Patients’ experiences with residents’ SDM performance are quite positive. This indicates that patients are satisfied with care and residents’ clinical performance even though the patients considered the relevance of SDM higher than the residents did and residents behaved rather directive from an observer perspective. However, the patients might have been unaware that their preferences mattered in decision-making, since residents hardly introduced this topic during most consultations.

Both residents and patients perceived the SDM performance of residents to be relatively effective compared to how performance was rated by observers. Although SDM-Q-Doc and SDM-Q-9 scores differ widely throughout literature, these positive perceptions are consistent with previous studies.^31–33^ The three instruments intend to capture different perspectives on SDM performance: observable behaviour and residents’ and patients’ experiences with the applied behaviour. Contrasting perceived and observed consultation performance, and learners becoming aware of potential incongruences, may be an important stimulus for fostering motivation to learn SDM. Although self-assessment of performance is often inaccurate, it may reveal learners’ unawareness of their level of competence to guide future learning.^34^

### Strengths and limitations

The main strength of this study is that we combined different approaches to describe residents’ SDM performance in actual consultations. Since there is no single-best instrument to measure SDM performance, we chose the validated instruments most relevant to our research questions.^18,21,23,25^ Combining OPTION^5^, SDM-Q-Doc and SDM-Q-9 may capture a broad perspective on residents’ performance.^18,19^ Since the three instruments intend to measure different constructs related to SDM, that is, observations of behaviour versus reported perceptions on performance, it is inherent that scores may differ between the instruments used.

To maximise the reproducibility of our findings, we trained observers comprehensively^24^ and indeed achieved a high degree of inter-observer reliability. We also tried to limit observer bias by randomizing the order in which consultations were scored. Hereby, we intended to minimize comparison between consultations and scores within one resident.

The results of this study showed residents’ SDM performance at group-level. We were not specifically focusing on individual performance nor did we perform multivariate analyses. However, when the data were analysed clustered per resident this did not yield different results. Our study was performed in four GP training institutes to increase the generalizability of our results.

An important limitation of our study is possible selection bias. Residents with a more positive attitude toward SDM and more critical attitude toward their own performance and learning might have been more likely to participate. We consider that our results underestimate the actual extent to which GP residents are incompetent. Accordingly, the gap between desired and actual performance is probably larger than we described. In addition, the residents were aware that we studied SDM. Therefore, they might have focused more on applying SDM during the study compared to their regular clinics, causing an overestimation of their observed SDM performance. Although residents mentioned that they also make an extra effort to practice SDM when being observed,^16^ the effect of recording their performance might be limited as residents are used to video-record their consultations.^35^ We recorded a complete clinic during the visit to the residents’ general practice. By doing so, we prevented residents’ selection of best example recordings and included a realistic representation of consultations in daily practice. Finally, data collection took place between 2017 and 2019.

### Implications for educational practice

Our findings suggest that more focused and consistent attention should be paid to SDM during medical residency. Feedback based on actual recorded consultations of residents with their patients during educational training sessions can support the development of SDM proficiency. This method might be useful to increase awareness of the need to learn to perform SDM, since residents’ self-perception may reflect a more positive picture compared to performance from an observer perspective. Self-monitoring during consultations and self-assessment of SDM performance after consultations may also direct the development of SDM skills, including how to tailor the phases to an individual patient in a particular consultation.^26,36^ Furthermore, observed or recorded consultations provide a starting point for reflection on attitudes toward (the relevance of) SDM and gaps in SDM knowledge and skills. Our findings also suggest that training should pay attention to recognizing the relevance of SDM, which may range from ‘small’ to complex decisions including the knowledge about potential options. Furthermore, the aforementioned contextual factors that might come into play need to be addressed in learning SDM. Additionally, training should also focus on how to support patients though the SDM process, adjusted to the preferred role of the individual patient. Since residents’ learning mainly takes place at the workplace, supervisors should be equipped with didactic competencies to support residents’ reflection on SDM, by improving their own SDM performance to become a good role model and coach.

## Conclusion

This study showed that there is room for increasing awareness of the potential incongruence between observed and perceived SDM performance during medical residency, in order to facilitate the implementation of SDM in clinical practice. Attention needs to be paid to how to tailor the process of SDM in a context sensitive manner throughout consultations, as well as residents’ beliefs and motivation towards performing SDM, using observation and external feedback on actual performance to integrate SDM in workplace learning.

## Supplementary Material

cmad125_suppl_Supplementary_Material

## Data Availability

The data that support the findings of this study are available from the corresponding author, AB, upon reasonable request.
