# Supplementary material for: Shared decision-making performance of general practice residents: an observational study combining observer, resident, and patient perspectives
Source: Fam Pract. 2024 Jan 11;41(1):50–9. doi: 10.1093/fampra/cmad125 (PMC10901472; doi:10.1093/fampra/cmad125)
Supplement: cmad125_suppl_Supplementary_Material [file cmad125_suppl_supplementary_material.pdf]

Appendix A: Characteristics of shared decision-making training at the four general practice specialty training institutes included in this study, for a study of shared decision-making performance in medical residency, 2017-2019

- Part of one or two stand-alone communication training sessions during formal training days in year 1 and/or 3
- Focus on training of SDM as a communication skill, generally without integration of medical knowledge and evidence based medicine skills
- No explicit integration in workplace learning

Appendix B: Associations between resident, patient and consultation variables on observed shared decision-making performance of general practice residents using OPTION[5] (n=98), for a study of shared decision-making performance in medical residency, 2017-2019

	Pearson correlation r-value[a] or independent sample t-test t-value[b] or one-way ANOVA F-value[c]	p-value
**Resident-level variable**		
Age	-0.006[a]	0.957
Gender	1.366[b]	0.175
Training year	0.359[b]	0.720
Training institution	0.861[c]	0.464
Clinical experience before entering GP training (setting)[d]	0.722[c]	0.489
Clinical experience before entering GP training (duration in months)	-0.033[a]	0.750
**Patient-level variable**		
Age	0.178[a]	0.090
Gender	0.947[b]	0.346
Educational level[e]	0.046[c]	0.955
**Consultation-level variable**		
Relevance of SDM according to resident[f]	4.571[b]	<0.001
Relevance of SDM according to patient[g,h]	not analysed	not analysed
Resident perception of SDM (SDM-Q-Doc)	0.512[a]	<0.001
Patient perception of SDM (SDM-Q-9)	0.214[a]	0.048
Duration of the consultation (minutes)	0.390[a]	<0.001
Initial or follow-up consultation	0.714[b]	0.477
Known or unknown patient	0.731[b]	0.467

Abbreviations: GP = general practice; SDM = shared decision-making

[a]Pearson correlation

[b]Independent sample t-test

[c]One-way ANOVA

[d]Setting of clinical experience before entering GP training is grouped in hospital setting only, non-hospital setting only and both hospital and non-hospital setting

[e]Educational level of patients is grouped in low (no education, primary education, lower secondary education), middle (higher secondary education, technical/vocational further education) and high (bachelor, master)

[f] SDM relevance was grouped in not relevant (score 0, 1 or 2) and relevant (score 3, 4 or 5) on the question 'how relevant was SDM in this consultation'

[g] SDM relevance was grouped in not relevant (score 0, 1 or 2) and relevant (score 3, 4 or 5) on the question 'for me it was important to be involved in the decision made in this consultation'

[h]The association between mean total OPTION[5] score of consultations in which SDM was relevant versus not relevant according to the patient was not analyzed, since only 6 consultations were found not relevant

Appendix C: Characteristics and number of patient inclusions per participating general practice resident (n=20) for a study of shared decision-making performance in medical residency, 2017-2019

Resident	Sex (male/female)	Age (years)	Location of GP training institute	GP training year	Clinical experience before entering GP training (months)	Specialty of clinical experience before entering GP training (months)	Practice setting	Total video-recorded consultations (n)
1	f	31	Maastricht	3	28	Psychiatry (6)<br>Surgery (16)<br>Elderly care medicine (6)	Urban, solo	3
2	m	29	Maastricht	1	24	Cardiology (12)<br>Neurology (12)	Rural, group	5
3	f	25	Maastricht	1	6	Elderly care medicine (6)	Urban, health center	4
4	f	29	Nijmegen	1	36	Emergency medicine (24)<br>Intensive care (12)	Rural, duo	4
5	f	26	Maastricht	1	24	Paediatrics (12)<br>Rehabilitation medicine (12)	Urban, health center	4
6	f	29	Maastricht	3	19	Neurology (12)<br>Elderly care medicine (7)	Urban, health center	6
7	f	28	Maastricht	3	12	Insurance medicine (12)	Urban, health center	8
8	f	31	Amsterdam	3	26	Internal medicine (11)<br>Emergency medicine (9)<br>Surgery (6)	Urban, health center	8
9	f	34	Amsterdam	3	48	Gynaecology (24)<br>Surgery (12)<br>Internal medicine (6)<br>Tropical medicine (6)	Urban, solo	5
10	f	35	Amsterdam	3	48	Gynaecology (48)	Urban, solo	4
11	f	35	Amsterdam	1	44	Internal medicine (18)<br>Elderly care medicine (12)<br>Rehabilitation medicine (8)	Urban, solo	3

						Psychiatry (6)		
12	m	25	Leiden	1	12	Elderly care medicine (12)	Urban, solo	4
13	f	28	Nijmegen	1	33	Elderly care medicine (24)<br>Emergency medicine (9)	Rural, health center	4
14	f	30	Amsterdam	1	36	Internal medicine (12)<br>Psychiatry (24)	Urban, health center	6
15	f	34	Leiden	3	52	Tropical medicine (48)<br>Physician for drivers medical examination (4)	Urban, group	4
16	f	28	Nijmegen	1	24	Elderly care medicine (12)<br>Emergency medicine (6)<br>Psychiatry (6)	Urban, health center	4
17	f	28	Leiden	1	24	Neurology (12)<br>Internal medicine (12)	Urban, group	4
18	f	30	Leiden	3	24	General hospital care (24)	Urban, health center	7
19	m	30	Leiden	1	30	Paediatrics (13)<br>Elderly care medicine (17)	Urban, health center	4
20	m	34	Nijmegen	3	60	Urology (42)<br>Intensive care (6)<br>Elderly care medicine (12)	Urban, health center	6
